# Building a pangenome alignment index via recursive prefix-free parsing

**DOI:** 10.1016/j.isci.2024.110933

**Published:** 2024-09-12

**Authors:** Eddie Ferro, Marco Oliva, Travis Gagie, Christina Boucher

**Affiliations:** 1 Department of Computer and Information Science and Engineering, Herbert-Wertheim College of Engineering, University of Florida, Gainesville, FL 32607, USA; 2 Faculty of Computer Science, Dalhousie University, Halifax, NS, Canada

**Keywords:** Bioinformatics, Biocomputational method, Classification of bioinformatical subject, Genomic analysis

## Abstract

Pangenomics alignment offers a solution to reduce bias in biomedical research. Traditionally, short-read aligners like Bowtie and BWA indexed a single reference genome to find approximate alignments. These methods, limited by linear-memory requirements, can only index a few genomes. Emerging pangenome aligners, such as VG, Giraffe, and Moni, address this by indexing more genomes. VG and Giraffe use a variation graph, while Moni indexes sequences accounting for repetition using prefix-free parsing to build a dictionary and parse. The main challenge is the parse’s size, which becomes significantly larger than the dictionary. To scale Moni, we propose removing the parse from the construction of the run-length encoded BWT (RLBWT), suffix array, and Longest Common Prefix (LCP) by applying prefix-free parsing recursively. This approach improves construction time and memory requirements, enabling efficient construction of RLBWT, suffix array, and LCP for large pangenomes, such as those from the Human Pangenome Reference Consortium.

## Highlights

• RPFP proposes a scalable method for constructing the RLBWT, Suffix Array, and LCP
• Provides a solution to the PFP parse size scaling poorly as the input size increases
• RPFP outperforms Big-BWT in time and memory on larger pangenomes
• Enables index construction that scales better for larger datasets, like 1kgp and HPRC

## Graphical abstract

**Figure:**
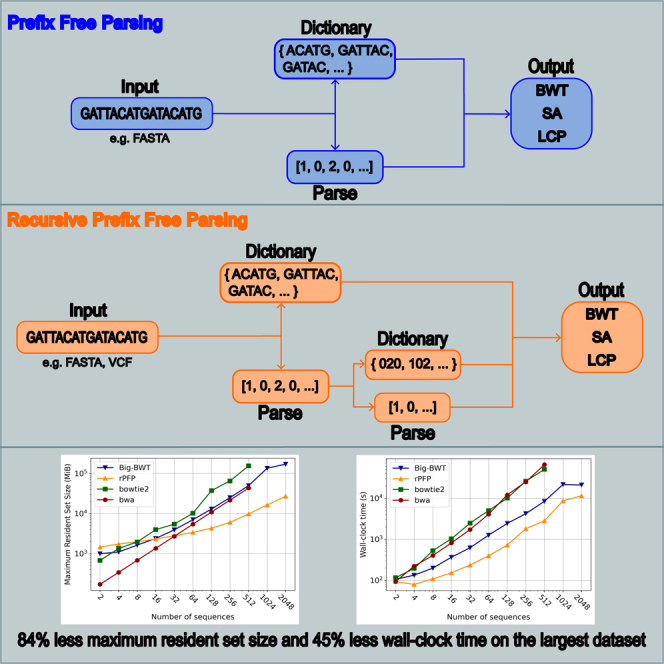

## Introduction

Read aligners have been fundamental to the analysis of countless datasets, including the 1,000 Genome Project,^1^ the 100K Genome Project,^2^ the 1,001 Arabidopsis Genomes project,^3^ and the Bird 10,000 Genomes (B10K) Project.^4^ They have enabled the discovery of genetic markers that have causal relationships with countless diseases and phenotypes. These methods take as input a set of sequence reads and a reference genome, build an index from the reference genome, and use this index to find alignments with the limitation that few insertions and deletions are allowed. Although short-read aligners—such as BWA^5^ and Bowtie^6^—are sufficient for aligning to a single or small number reference genome(s), they are unable to index the data of an entire population. To understand why, it is necessary to consider the underlying data structure used for indexing the genomes, which is the FM-index.^7^ This data structure combines the Burrows-Wheeler transform (BWT)^8^ array, the suffix array (SA), as well as some smaller auxiliary data structures (i.e., those used to support rank queries). The FM-index requires linear space with respect to the input for its construction and storage and thus, is difficult to construct for large, terabyte-sized datasets.

Nonetheless, there exists a small handful of solutions for indexing and aligning to a pangenome, including Giraffe,^9^ VG,^10^ and Moni.^11^ These methods follow different paradigms for pangenomics alignment; Giraffe^9^ and VG^10^ build and index a graph from a multiple alignment of the genomes. Moni^11^ indexes all the reference genomes by taking advantage of the repetition in the reference genomes. Yet, even with these advancements, there still exists a critical gap in our ability to index the increasing number of publicly available genomes for alignment. We make progress toward closing the gap by developing an algorithm for indexing 1,000 diploid haplotypes in less than 1TB of memory without any preprocessing. The Prefix-free parsing (PFP) algorithm is fundamental to this indexing, which takes as input one or more sets of strings (genomes), and two integers *w* and *p*. It then uses a rolling hash to find all *w*-length substrings that have hash values equal to zero, i.e., hmodp=0. These *trigger strings* are then used to define a parse: each substring of the input string that begins and ends at a trigger string defines a substring of the parse. All unique substrings in the parse are stored in a dictionary that is sorted lexicographically, which allows the parse to be stored as a list of integers (rank of the substring in the dictionary). Rossi et al.^11^ showed that PFP can build an index that can efficiently find maximal exact matches (MEMs) between a set of reads and reference genome(s). This index consists of a run-length compressed BWT (RLBWT), sampled Suffix Array (SA), and sampled Longest Common Prefix (LCP) array that stores the longest common prefix between subsequent rotations in the BWT. These MEMs can be extended to find full alignments between the reads and genome(s).

While PFP performs effectively in practical applications, its scalability can be enhanced further by compressing the parse during the construction. This is because, even though the dictionary size remains manageable for large, repetitive inputs, the expansion of the parse is not insignificant. For example, for between 200 and 2,400 human haplotypes of chromosome 19, the dictionary size increased by 0.5 GB but the parse increased by more than 10 GB. In light of these insights, Oliva et al.^12^ presented recursive PFP which applies PFP to the parse, which leads to a dictionary and parse of the parse. These resulting data structures are substantially smaller than the original parse but lead to an algorithmic challenge of constructing the RLBWT, SA, and LCP without access to the information contained in the parse. Oliva et al.^12^ showed how the RLBWT can be built from recursive PFP but left the construction of SA, and LCP open. In this paper, we address this open problem by giving algorithms to construct the SA and LCP—along with the BWT—using recursive PFP. More formally, given an input string *T*, the prefix-free parse of *T* (i.e., $D_{T}$ and $P_{T}$), and the prefix-free parse of P (i.e., $D_{P}$ and $P_{P}$), we give a constructive proof that shows the RLBWT, SA, and LCP can be constructed in $O\left(\left|D_{T}\right|+\left|D_{P}\right|+\left|P_{P}\right|\right)$ working space, removing $\left|P_{T}\right|$ from the construction space. Hence, from a practical perspective this demonstrates how to efficiently build a pangenome index, and from a theoretical perspective this work contributes to the efficient construction of compressed data structures, which includes construction of the BWT^13^^,^^14^^,^^15^^,^^16^ and SA.^17^^,^^18^^,^^19^

Lastly, we evaluated the performance of rPFP compared to Bowtie2, BWA, and Big-BWT on constructing indices for various datasets. Our evaluation included pangenomic datasets with diploid sequences of chromosome 19 from the 1,000 Genomes Project, increasingly larger collections of SARS-CoV-2 genomes from the NCBI Data Hub, and assemblies from the Human Pangenome Reference Consortium (HPRC)^20^ Year 1 version 2 data freeze. rPFP consistently outperformed Big-BWT in terms of wall-clock time, requiring significantly less memory for larger datasets. On smaller datasets, Big-BWT sometimes performed better with regards to memory usage due to the increased overhead of rPFP for computing the SA and LCP. For the SARS-CoV-2 data, rPFP was the fastest and most memory-efficient method, while Bowtie2 was the slowest and most memory-intensive. Only rPFP and Big-BWT were able to construct indices for all tested datasets from the Human Pangenome Reference Consortium within the given parameters, with rPFP demonstrating less memory usage and wall-clock time as the dataset size increased. Overall, rPFP proved to be highly efficient and scalable for large genomic datasets.

## Results

### Overview of rPFP

rPFP constructs the RLBWT, SA, and LCP using recursive PFP which applies PFP to the parse, leading to a dictionary and parse of the parse. These resulting data structures are substantially smaller than the original parse, reducing the memory requirement for construction. We implemented rPFP in ISO C++ 20. We used the sdsl library for rank and select support^21^ and gSACA-k to compute the SA in our data structures.^22^ We designed three sets of experiments to evaluate the performance of rPFP: (1) We compare rPFP with Bowtie2, BWA, and Big-BWT on 25k, 50k, 100k, 200k, and 400k concatenated copies of the SARS-CoV 2 virus fasta; (2) We compare rPFP with Bowtie2,^6^ BWA^5^ and Big-BWT on 2, 4, 8, 16, 32, 64, 128, 256, 512, 1024 and 2048 diploid sequences of chromosome 19 from the 1,000 Genomes Project; (3) We select the methods that are able to successfully build their index on chr19.2048 in less than 24 h of wall clock time and tested those methods on 1, 2, 4, 8, and 16 assemblies from the Human Pangenome Reference Consortium (HPRC) Year 1 version 2 data freeze.^20^ For the chromosome 19 experiment, since Bowtie 2 and BWA only take a FASTA file as input, we created a FASTA from the VCF files containing the variations. We remark that PFP can work directly from the VCF file.^23^ In reporting the results of all experiments, we do not take into account the resources needed to create the FASTA files and otherwise prepare the datasets. The results for rPFP and Big-BWT include the time needed to compute the parse and the dictionary. The SARS-CoV-2 and Chr19 experiments were run on a node utilizing 32 cores and 300 GB of memory with no other significant tasks running and a time limit of 24 h of wall clock time. The HPRC experiments were run on a node utilizing 32 cores and 700 GB of memory with a limit of 36 h of wall clock time. All methods were run with their default parameters using mult-threading whenever possible. We note that Big-BWT constructs the full LCP while rPFP constructs the sampled LCP. Additionally, when the input is compressed, Big-BWT cannot perform multi-threading. We consider this a limitation of the software as pangenomic applications require the dataset to be compressed due to the size of the files.

### Results on SARS-CoV-2 data

**Figure 1:**
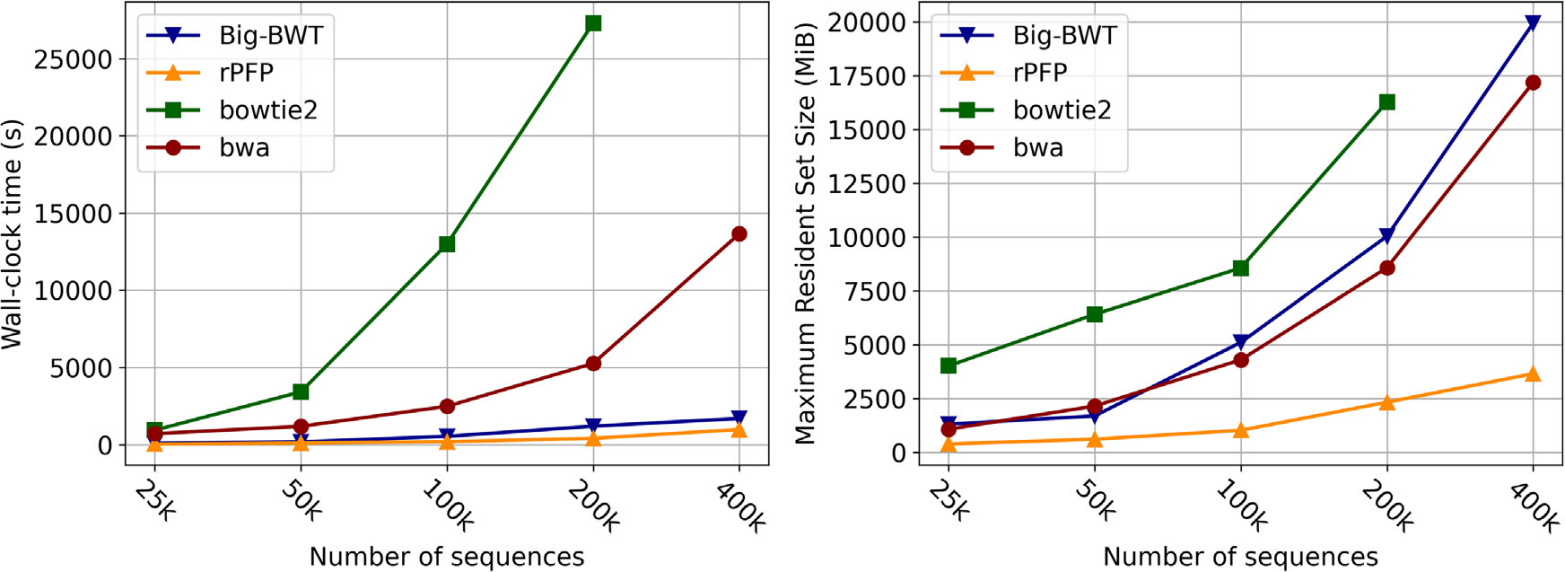
Results on SARS-CoV-2 datasets Index construction wall clock time in seconds (left) and peak memory in MiB (right).

We conducted an experiment on multiple copies SARS-CoV-2 virus complete genome to show results on highly repetitive data. This was done on increasingly larger collections of the SARS-CoV-2 genomes that were taken from the Covid 19 Data Portal.^24^ These datasets were constructed by appending different copies of the full genome to a fasta file to reach the desired size. Starting with a dataset of 25 thousand copies of the genome with each sequential dataset doubling the number of copies until 400,000 copies is reached. We refer to these subsets as sars.25k,…,sars.400k. The wall clock time and maximum resident set size plots for this experiment can be seen in Figure 1. We note that all methods were capable of constructing their index on sars.25k in a comparable amount of time. The fastest indexing time was rPFP at 44 s, which is half the time it took Big-BWT to construct the index. Additionally, rPFP only took a maximum of 391 MiB, which is less than a quarter of the 1310 MiB that Big-BWT required. The slowest was Bowtie2, requiring 935 s to index, with BWA taking a little less at 712 s. Bowtie2 also required the most amount of memory at 4016 MiB. The trend in performance remains consistent as the input size increases. Bowtie2 was unable to compute the index on sars.400k within the 24-h time limit. At sars.400k, rPFP took 60% of the time it took Big-BWT to construct the same index and only required 18% of the memory that Big-BWT required. BWA took the most amount of time, but took 2.89 GB less than Big-BWT. We expected Big-BWT to outperform rPFP on smaller datasets; the fact that it doesn’t is likely due to the difference in rPFP computing a sample of the SA and LCP compared to Big-BWT computing the full SA and LCP. However, as the dataset size increases, the difference in time and memory is too large to be attributed to just the different SA and LCP calculations.

### Results on Chromosome 19

**Figure 2:**
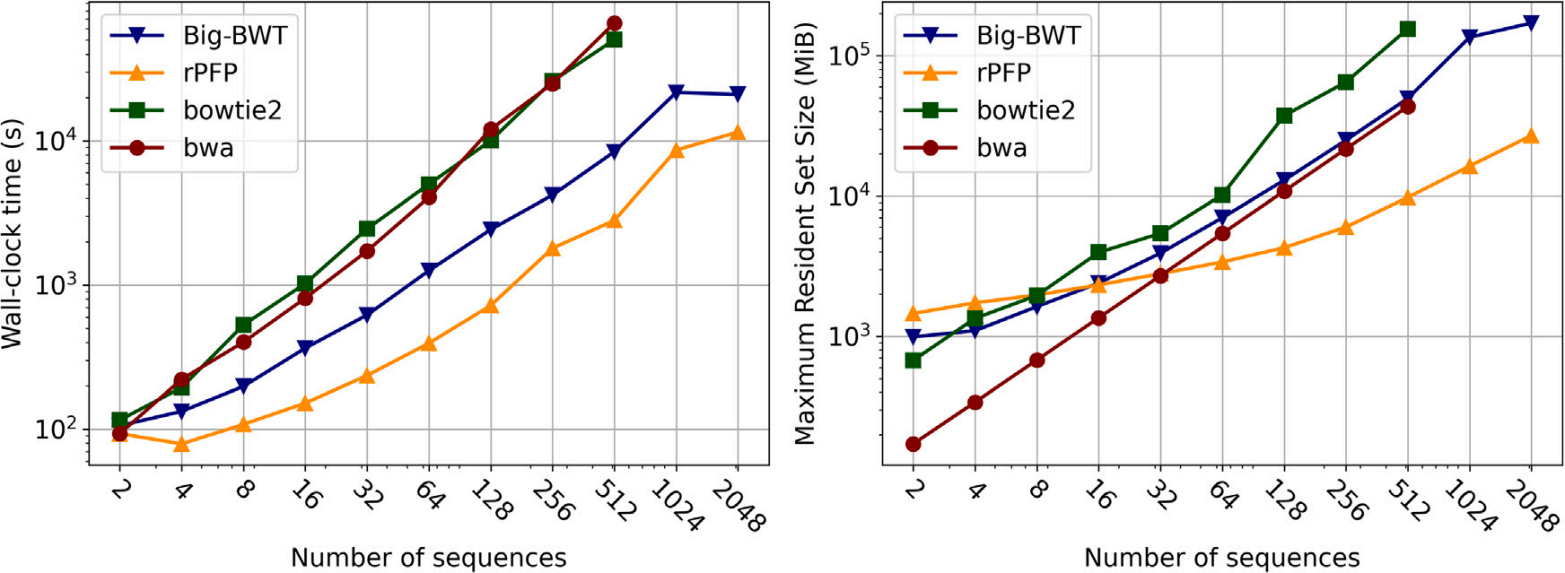
Results on Chromosome 19 datasets Index construction wall clock time in seconds (left) and peak memory in MiB (right).

We first focused on Chromosome 19 from the 1,000 Genomes Project, generating two haplotypes for each diploid sequence. We refer to the subsets as chr19.2,…,chr19.2048. The wall clock time and maximum resident set size plots for this experiment can be seen in Figure 2. We note that Bowtie2 and BWA were only capable of indexing up to chr19.512 before exceeding the time and memory constraints of the experiment. We found that rPFP performs better at every subset in terms of wall clock time and requires less memory for the larger datasets. For chr19.2, rPFP requires 93 s which is the same amount of time required by BWA. In contrast, Big-BWT and Bowtie2 needed an additional 20 s complete the index for the same dataset. However, it does require 463.23 MiB more than the second most memory consuming method at that dataset. For chr19.8, rPFP requires 14 MiB more then Bowtie2 and in larger subsets rPFP requires significantly less than Bowtie2. For chr19.16, rPFP requires 70 MiB less than Big-BWT, but this gap continues to increase as the size of the dataset increases. Then, for chr19.32, rPFP requires only 85 MiB more than BWA and requires less than BWA afterward. At chr19.2048, rPFP requires only 55% of the time and 16% of the memory that Big-BWT requires. The noticeable trend is that as the size of the input increases rPFP’s memory and time requirement grows at a slower rate than the other methods.

### Results on human Genome

**Figure 3:**
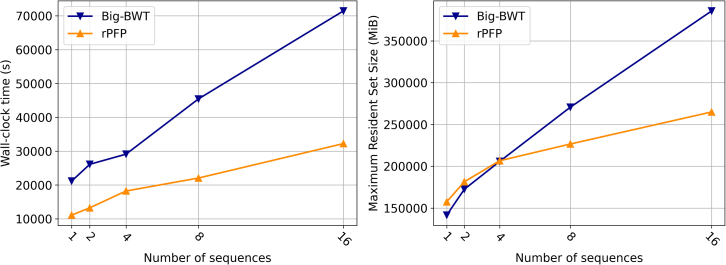
Results on Human Pangenome datasets Index construction wall clock time in seconds (left) and peak memory in MiB (right).

We evaluated the construction of the BWT, SA, and LCP on a pangenome from HPRC assemblies using rPFP and Big-BWT, as they were the only ones capable of building the index on chr19.2048 within the experiment constraints. We constructed increasingly larger datasets containing assemblies from the HPRC Year 1 version 2 data freeze. These datasets were constructed by concatenating the maternal and paternal assemblies for each sample to a single copy of the GRCh38 reference. Each dataset doubled the number of samples of the previous dataset. We refer to these as hprc.1,…,hprc.16. The wall clock time and maximum resident set size plots for this experiment can be seen in Figure 3. Both methods were given 32 cores, 700 GB of memory, and 36 h to construct the index, which they were able to do for all datasets. For hprc.1, rPFP was able to construct the index in 3 h and 4 min, compared to Big-BWT, which required 5 h and 52 min. However, rPFP took 17 GB of memory more than Big-BWT to construct the index for hprc.1. At hprc.4, which rPFP required 217 GB which is marginally larger than Big-BWT’s 216 GB requirement. For larger datasets, rPFP requires significantly less memory that Big-BWT needs, only needing 69% of the memory Big-BWT needs for hprc.16. We again expect rPFP to require more time than Big-BWT for the smaller of the datasets, but Big-BWT likely takes longer in this instance due to the lack of multithreading because the datasets were compressed. However, we note that as the input size increases, the time and memory required by rPFP grows at a significantly smaller rate compared to Big-BWT. This indicates that we likely outperform Big-BWT in larger datasets, even if the input were not compressed, which is necessary due to the size.

### Results on recursive PFP compression

**Figure 4:**
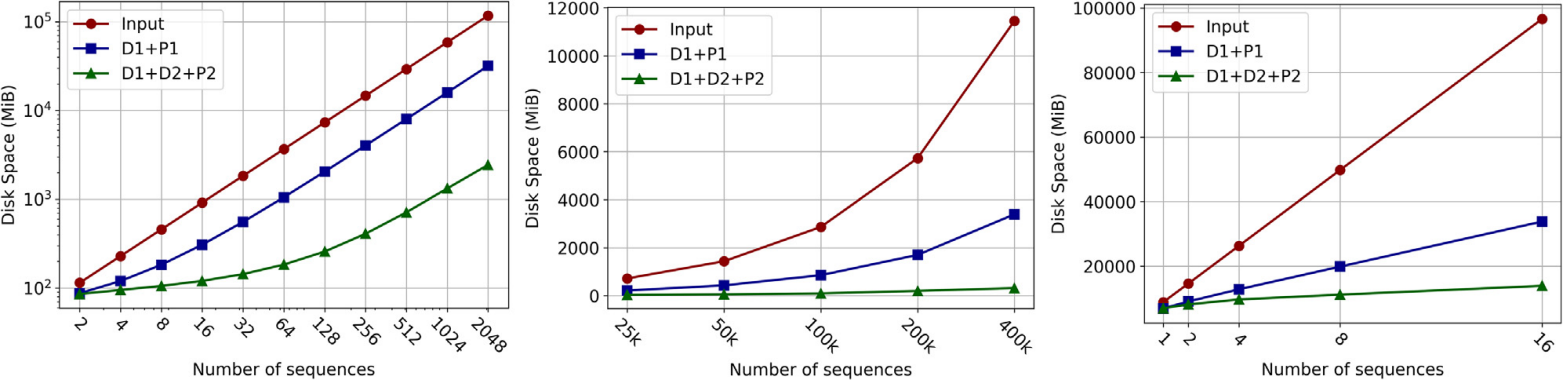
Disk size of the input, PFP, and rPFP for the Chr19 experiments (left), the SARS-CoV2 experiments (middle), and the HPRC experiments (right)

We briefly discuss the compression of recursive PFP as it relates to the original input and PFP. The required disk space for the input, PFP, and recursive PFP as the number of sequences increases is shown in Figure 4. It is well known that PFP significantly reduces the disk space required to store the input compared to storing just the input. The improvement that recursive PFP provides over PFP is not immediate. At chr19.2, recursive PFP only reduces the size by 2 MiB and at hprc.1 recursive PFP takes 54 MiB more. Again, as the size of the input increases, the compression of recursive PFP becomes more apparent. For the Chromosome 19 experiments, starting at chr19.16, recursive PFP starts to take less than 50% of the space required by PFP and at chr19.2048 recursive PFP takes only 7.7% of the space that PFP requires to store the input. The HPRC experiments show similar results in that recursive PFP takes up only 41% of the space that PFP takes up. In the SARS-CoV-2 datasets, as we increase the number of sequences, we do not add a lot of new genetic data as we only add similar copies of the same genome. This means that the size of the dictionary does not increase by much when the input does, only the parse does, which recursive PFP is designed to reduce. We can see as much as recursive PFP never takes more than 15% of the space that PFP takes for any size dataset in the SARS-CoV2 experiment.

## Discussion

We developed an efficient algorithm for constructing the SA and LCP concurrently with the BWT that eliminates the need for the parse from PFP in the construction process. For increasingly large, repetitive input the size of the parse grows significantly more than the size of the dictionary; thereby, eliminating the parse significantly increases the space efficiency of the construction, which is the bottleneck in the construction for large repetitive input. Recursively running PFP on the parse is the algorithmic contribution that enables us to achieve these gains. Removing the parse creates the algorithmic puzzle of piecing together the construction of the SA and LCP via smaller auxiliary data structures that nontrivially take the place of the parse. This innovation slashes the memory usage by 2.7 times when applied to extensive collections of chromosome 19. Furthermore, Bowtie2 and BWA were unable to index beyond 256 copies of chromosome 19. The efficiency of our method becomes even more striking with full human genomes, where our approach requires less time and memory than regular PFP.

### Limitations of the study

A limitation of our study is that each method, including our own, constructs a different index and imposes unique restrictions on input data. These differences can hinder direct comparisons between competing methods. Future research should explore simpler compression methods and the practicality of continued recursion to optimize memory usage and computational efficiency in data compression and sequence analysis. Investigating whether the information contained by $P_{T}$ can be compressed by simpler means, such as delta-encoding, is a promising direction. Delta-encoding, which stores differences between sequential data points, may offer significant space savings if the data exhibits low variability. Future work could include empirical evaluations to compare delta-encoding with recursive parsing, developing new algorithms leveraging delta-encoding for phrase occurrences in $D_{T}$, and exploring hybrid methods that balance simplicity and efficiency. Additionally, the concept of continued recursion to parse $P_{T}$ and beyond, presents an intriguing research avenue. We hypothesize that there is limited benefit and increased complexity, but this assumption warrants rigorous testing. Future research could involve theoretical analyses to understand the trade-offs, experimental validations to measure performance metrics like compression ratio and resource utilization, and the design of efficient auxiliary data structures for recursive parsing. Addressing these questions will enhance theoretical and practical understanding, potentially leading to more efficient and practical compression solutions for pangenomic datasets.

## Resource availability

### Lead contact

Further information and requests for resources should be directed to and will be fulfilled by the first author Eddie Ferro at eferro1@ufl.edu.

### Materials availability

This study did not generate new unique reagents.

### Data and code availability

• Data: For the experiments on Chromosome 19, the dataset was constructed using samples from publicly available data from the 1,000 Genomes Project that can be downloaded at http://ftp.1000genomes.ebi.ac.uk/vol1/ftp/phase3/data/. For the experiments on full Human Genomes, the dataset was constructed using samples from the Human Pangenome Reference Consortium that is publicly available at https://github.com/human-pangenomics/HPP_Year1_Assemblies. Both experiments also used the GRCh38 assembly that can be found at https://www.ncbi.nlm.nih.gov/datasets/genome/GCF000001405.40/. The SARS-CoV-2 dataset was constructed using genomes publicly available at https://www.covid19dataportal.org.
•Code: Our code is publicly accessible at https://github.com/EddieFerro/rPFP.
•Other: Any additional information required to reanalyze the data reported in this paper is available from the lead contact upon request.

## Acknowledgments

This research was funded by 10.13039/100000002NIH/10.13039/100000060NIAID grant R01AI14180, NSF/BIO grant DBI-2029552 and NSF/SCH grant INT-2013998 to C.B., and 10.13039/100000002NIH/10.13039/100000051NHGRI grant R01HG011392 to Ben Langmead.

## Author contributions

Conceptualization was completed by C.B. and T.G.; Methodology was completed by all authors; Formal Analysis was completed by all authors; Implementation was done by E.F. and M.O.; Visualization was completed by E.F. and C.B.; Supervision was by CB; Writing and editing was done by all authors.

## Declaration of interests

The authors declare no competing interests.

## STAR★Methods

### Key resources table

**Table:** 

REAGENT or RESOURCE	SOURCE	IDENTIFIER
**Deposited Data**		

1000 Genomes phase 3 release	1000 Genome Project Consortium (2015)^1^	http://ftp.1000genomes.ebi.ac.uk/vol1/ftp/phase3/data/
Sars-CoV-2 Assembly Collection	Boucher et al.,2021^25^	https://www.covid19dataportal.org
GRCh38 assembly	Church et al.,2015^26^	https://www.ncbi.nlm.nih.gov/datasets/genome/GCF_000001405.40/
HPRC Year 1 version 2 data freeze	Liao et al.,2023^20^	https://github.com/human-pangenomics/HPP_Year1_Assemblies

**Software and algorithms**		

rPFP	This paper	https://github.com/EddieFerro/rPFP
sdsl	Gog et al.2014^27^	https://github.com/simongog/sdsl-lite
BWA	Li et al.2013^5^	https://github.com/lh3/bwa
Bowtie2	Langmead et al.2012^6^	https://github.com/BenLangmead/bowtie2
pfp-thresholds	N/A	https://github.com/maxrossi91/pfp-thresholds
Big-BWT	Boucher et al.2019^28^	https://gitlab.com/manzai/Big-BWT

### Method details

#### Preliminaries

##### String notations

A string *T* is a finite sequence of symbols T=T[1..n]=T[1]⋯T[n] over an alphabet $\Sigma =\left\{c_{1},\ldots ,c_{\sigma }\right\}$ whose symbols can be unambiguously ordered. We denote ε as the empty string, |T| as the length of *T*, and $c^{k}$ as the string formed by the character *c* repeated *k* times.

We denote by T[i..j] the substring T[i]⋯T[j] of *T* starting at position *i* and ending in position *j*, with T[i..j]=ε if i>j. For a string *T* and 1≤i≤n, T[1..i] is called the *i*-th prefix of *T*, and T[i..n] is called the *i*-th suffix of *T*. We call a prefix T[1..i] of *T* a *proper prefix* if 1≤i<n. Similarly, we call a suffix T[i..n] of *T* a *proper suffix* if 1<i≤n. Given a set of strings S, S is *prefix-free* if no string in S is a prefix of another string in S. We denote by ≺ the lexicographic order: for two strings $T_{2}\left[1..m\right]$ and $T_{1}\left[1..n\right]$, $T_{2}≺T_{1}$ if $T_{2}$ is a proper prefix of $T_{1}$, or there exists an index 1≤i≤n,m such that $T_{2}\left[1..i-1\right]=T_{1}\left[1..i-1\right]$ and $T_{2}\left[i\right]<T_{1}\left[i\right]$. We define co-lexicographic order as the lexicographic order obtained by reading $T_{1}$ and $T_{2}$ from right to left instead of from left to right.

##### Suffix array and burrows wheeler transform

Given a string T[1..n], the *suffix array*,^29^ denoted by ${\text{SA}}_{T}$, is the permutation of {1,…,n} such that $T\left[{\text{SA}}_{T}\left[i\right]..n\right]$ is the *i*-th lexicographically smallest suffix of *T*. We refer to ${\text{SA}}_{T}$ as SA when it is clear from the context. The Burrows-Wheeler transform of a string T[1..n], denoted by ${\text{BWT}}_{T}$, is a reversible permutation of the characters in *T*.^8^ If we assume *T* is terminated by a special symbol $ that is lexicographically smaller than any other symbol in Σ, we can define ${\text{BWT}}_{T}\left[i\right]=T\left[{\text{SA}}_{T}\left[i\right]-1\;\mathrm{mod}\;n\right]$ for all i=1,…,n.

We denote the *inverse suffix array* as ${\text{ISA}}_{T}$, and define it as ${\text{ISA}}_{T}\left[{\text{SA}}_{T}\left[i\right]\right]=i$ for all i=1,…,n. We refer to ${\text{ISA}}_{T}$ as ISA when it is clear from the context.

##### Longest common prefix

Given two strings S[1..n] and T[1..m] we refer to the *longest common prefix* as the substring S[1..l]=T[1..l] such that l=maxi∣S[1..i]=T[1..i]. We denote the length l of the longest common prefix of *S* and *T* as lcp(S,T). Given the SA of *T*, we define the *longest common prefix array* of *T* as an array of length *n* such that it stores the length of the longest common prefix between all consecutive pairs on suffixes in SA. We denote this as LCP. Hence, we have LCP[1]=0 and for i=2,..,n we have LCP[i]=LCP(T[SA[i−1]..n],T[SA[i]..n]). The range minimum query (RMQ) is a query that can be performed on a totally ordered set to find the minimum value in range in the array. When performed on the LCP array, the minimum value returned is the length of the LCP of the suffix at the start of the range and the suffix at the end of the range. In practice, since the LCP array is static and the RMQ is performed often, it is more efficient to preprocess the RMQ and store it in a data structure that is comparable in size to the LCP array.

##### Overview of prefix-free parsing

PFP takes as input a string *T* of length *n*, and two integers greater than 1, which we denote as *w* and *p*. It produces a parse of *T* consisting of overlapping phrases, where each unique phrase is stored in a dictionary in their lexicographic ordering. The parse is built by storing the order the dictionary phrases appear in the original input text by their rank in the dictionary. We denote the dictionary as D and the parse as P. We note that we refer to D as a dictionary, but in practice it is stored as a string with all phrases concatenated together with a special symbol separating them. As the name suggests, the output produced by PFP has the property that none of the suffixes of length greater than *w* of the phrases in D is a prefix of any other. We formalize this property through the following lemma. We refer to PFP of *T* as PFP(T), consisting of the dictionary D and the parse P.

##### Lemma 1

^28^*If we are given a string T and*PFP(T)*then the set*S*of distinct proper phrase suffixes of length at least w of the phrases in* D *is a prefix-free set.*

The first step of PFP is to append *w* copies of $ to *T*, where $ is a special symbol lexicographically smaller than any element in Σ. For the sake of the explanation, we consider the string $T^{\prime }={\$}^{w}T{\$}^{w}$^1^. Next, we characterize the set of trigger strings E, which define the parse of *T*. Given a parameter *p*, we construct the set of trigger strings by computing the Karp-Rabin hash, $H_{p}\left(t\right)$, of substrings of length *w* by sliding a window of length *w* over $T^{\prime }={\$}^{w}T{\$}^{w}$, and letting ∖E be the set of substrings $t=T^{\prime }\left[s..s+w-1\right]$, where $H_{p}\left(t\right)\equiv 0$ or $t={\$}^{w}$. This set E will be used to parse ${\$}^{w}T{\$}^{w}$. PFP can be used as a preprocessing step to build data structures such as the BWT, the SA, and the LCP.

Given the PFP of a string *T*, we first show how to build the BWT of *T* from D and P using workspace proportional to sum of the total length of P and the elements of D, and O(n)-time when we can work in internal memory. This is referred to as the Big-BWT algorithm, which we now briefly describe. To determine the relative order of the characters in the ${\text{BWT}}_{T}$—and hence, the relative lexicographic order of the suffixes following two characters in *T*—we start by considering the case in which two characters are followed in *T* by two distinct proper phrase suffixes α,β∈S. Hereon, when we refer to a proper phrase suffix, it is one that has length at least *w*. The following Corollary follows from Lemma 1.

##### Corollary 1

^28^*If two characters*T[i]*and*T[j]*are followed by different phrase suffixes* α *and* β*, where* |α|≥w *and* |β|≥w*, then* T[i] *precedes* T[j] *in the* BWT *of T if and only if* α≺β.

In other words, for some of the characters in ${\text{BWT}}_{T}$, it is sufficient to only consider the proper phrase suffixes which follow them in *T* to break the ambiguity. When this is not enough, we need the information contained in the parse.

##### Lemma 2

^28^*We let t and*$t^{\prime }$*be two suffixes of T that begin with the same proper phrase suffix* α*, and let q and* $q^{\prime }$ *be the suffixes of P that have the last w characters of those occurrences of* α *and the remainders of t and* $t^{\prime }$*. If* $t≺t^{\prime }$ *then* $q≺q^{\prime }$.

Next, we give some intuition on how these two lemmas are used to compute the ${\text{BWT}}_{T}$. We consider each proper phrase suffix α in S and compute the range in the BWT containing the characters immediately preceding in the string *T* the occurrences of α. In order to compute the range, we only need to know the starting position and length of the range. The starting position of the range for α is the sum of the frequencies in *T* (or P) of the proper phrase suffixes of length at least *w* that are lexicographically less than α. The length of the range is the frequency of α. Now suppose that we are working on the range of the BWT of *T* corresponding to the *i*-th proper phase suffix α. If all the occurrences of α in *T* are preceded by the same character c, then the range of the BWT of *T* associated with α will consist of all c’s. Therefore, no further computation is needed to define said range. Otherwise, the occurrences in the input of the *i*-th proper phrase suffix α are preceded by different characters then we make use of Lemma 2 to break the ambiguity. The order of the characters preceding α in *T* can be obtained from the order in which the phrases containing α appear in the BWT of P.

The algorithm for computing the BWT was expanded to compute the SA values along with the BWT.^30^ For each occurrence, $\alpha_{i}$, of a proper phrase suffix α∈S, we can obtain its relative order among the other proper phrase suffixes using Lemma 2. In order to associate to $\alpha_{i}$, its SA value, it is sufficient to store for each occurrence of each phrase $d_{j}\in D$ its ending position in *T*. We denote the array containing the ending positions of $d_{j}\in D$ as $EP_{j}$. Let us assume that $\alpha_{i}$ is stored in the *k*-th occurrence of the phrase $d_{j}\in D$, we are able to compute the associated SA value as $EP_{j}\left[k\right]-\left|\alpha_{i}\right|+1$. Storing all the EP arrays requires O(|P|) words. As described earlier, storing O(|P|) words can be a significant bottleneck for large repetitive datasets, and motivates the need for a BWT and SA construction algorithm that uses less than O(|P|) words. In the next section, we introduce our recursive algorithm to address this need.

#### Overview of recursive prefix-free parsing

We assume that PFP was run on the input string *T* with window size $w_{1}$ and integer $p_{1}$. We denote the set of trigger strings defined by $w_{1}$ and $p_{1}$ as $E_{1}$, and the output as $P_{T}$ and $D_{T}$. Next, we run PFP on the parse $P_{T}$ with window size $w_{2}$ and integer $p_{2}$. We denote the set of trigger strings defined by $w_{2}$ and $p_{2}$ as $E_{2}$, and the output of this step as $P_{P}$ and $D_{P}$. Next, we denote the set of proper phrase suffixes of length greater than $w_{1}$ of the phrases in $D_{T}$ as $S_{T}$ and, analogously, we denote the set of proper phrase suffixes of length greater than $w_{2}$ of the phrases in $D_{P}$ as $S_{P}$.

Here we define a meta-character to be any character in the $D_{P}$ phrases. Each unique meta-character represents a specific phrase from $D_{T}$, much like the values in $P_{T}$. In practice, these meta-characters are just the integers of $P_{T}$ converted to a character. For ease of understanding, we will now refer to phrases in $D_{P}$ as meta-phrases and their proper suffixes as proper meta-phrase suffixes.

Previously, Oliva et al. gave an $O\left(\left|D_{P}\right|+\left|D_{P}\right|+\left|P_{P}\right|\right)$-space construction algorithm,^31^ which we now briefly describe. Hereon, when we refer to a proper phrase suffix, we imply it is a proper phrase suffix of length at least $w_{1}$ or $w_{2}$. We let α be a proper phrase suffix in $S_{T}$, and characterize it as being one for the following types: (1) easy proper phrase suffixes; (2) hard-easy proper phrase suffixes; and (3) hard-hard proper phrase suffixes.

##### Theorem 1

^31^*Given a string T, the dictionary*$D_{T}$*and the parse*$P_{T}$*obtained by running* PFP *on T, and the dictionary* $D_{P}$ *and the parse* $P_{P}$ *obtained by running* PFP *on* $P_{T}$*, we can compute the* ${\text{BWT}}_{T}$ *from* $D_{T}$*,* $D_{P}$ *and* $P_{P}$ *using* $O\left(\left|D_{T}\right|+\left|D_{P}\right|+\left|P_{P}\right|\right)$ *workspace.*

##### Definition 1

We let α be a proper phrase suffix in $S_{T}$ that is in the set of phrases $D_{\alpha }=\left\{d_{i},\ldots ,d_{k}\right\}$, which is a subset of $D_{T}$. We define α to be an easy proper phrase suffix if and only if each phrase in $D_{\alpha }$ is preceded by the same character.

For example, we consider T= ##GATTACAT#GGATTAGAT## and suppose α is equal to ATTA, which is the set $D_{\alpha }=\left\{\#\#GATTA,GGATTA\right\}$. Since α is only preceded by a G in both rotations, the BWT of *T* will be G in both cases. This is is an example of an easy proper phrase suffix. As previously discussed, the parse is not needed for computing the BWT entries for easy proper phrase suffixes. Next, we consider our first case where the parse is needed to build the BWT.

##### Definition 2

We let α be a proper phrase suffix in $S_{T}$ that is in phrases $D_{\alpha }=\left\{d_{i},\ldots ,d_{k}\right\}$, and let $S_{\beta }\subset S_{P}$ be the set of proper meta-phrase suffixes that are preceded by any phrase in $D_{\alpha }$. We define α as a hard-easy suffix if and only if the following conditions hold: (a) there is at least one pair of phrases in $D_{\alpha }$ where the characters preceding α in these phrases are different; and (b) no pair of occurrences in $P_{T}$ of phrases in $D_{\alpha }$ are followed by the same proper meta-phrase suffix in $S_{\beta }$.

In other words, for hard-easy suffixes it is enough to look at the proper phrase suffix in $S_{P}$ to break the ambiguity. We label the remaining suffixes as hard-hard, which we define as follows.

##### Definition 3

We let α be a proper phrase suffix in $S_{T}$ that is in phrases $D_{\alpha }=\left\{d_{i},\ldots ,d_{k}\right\}$, and let $S_{\beta }\subset S_{P}$ be the set of proper meta-phrase suffixes that are preceded by any phrase in $D_{\alpha }$. We define α as a hard-hard suffix if and only if the following conditions hold: (a) there exists at least one pair of phrases in $D_{\alpha }$ in which the character preceding α is different; and (b) at least two phrases of $D_{\alpha }$ precede in $P_{T}$ the same proper meta-phrase suffix in $S_{\beta }$.

We illustrate these types of proper phrase suffixes in Figure S1. In order to calculate the BWT for these latter two cases, we define and use the following auxiliary data structures.

##### Definition 4

*We define a table*$T_{T}$*containing*$O\left(\left|S_{T}\right|\right)$*rows and*O(1)*columns, such that for each* α *in lexicographic order in* $S_{T}$*, we store its range in the* BWT *of T along with the co-lexicographic sub-range of the elements of* $D_{T}$ *which store the occurrence of* α*. That is, for each character* c*, the columns of* $T_{T}$ *store the range of co-lexicographically sorted phrases that end in* α *and have* c *in position* |α|+1 *from the end.*

An example of this is Table S1. In this table, we store all the proper phrase suffixes of the input text in S1 alongside the range they cover in the BWT and the co-lexicographic subrange of the characters that precede this proper phrase suffix in all the phrases that contain it. Next, we define the table $T_{P}$, and the grid G as follows.

##### Definition 5

We define a table $T_{P}$ containing $O\left(\left|S_{P}\right|\right)$ rows and O(1) columns, such that for each β in lexicographic order in $S_{P}$, we store in $T_{P}$ the co-lexicographic range of the meta-phrases of $D_{P}$ that contain β along with the meta-characters that precede β in $P_{T}$.

An example of this is Table S2. In this table, we store all the proper phrase suffixes of the original parse, which is not shown. For each of these proper phrases suffixes, we store all the meta-characters that precede it in any phrase and the co-lexicographic range of the phrases that each suffix belongs to in the grid G.

##### Definition 6

*We define the grid*G*containing*$O\left(\left|P_{P}\right|\right)$*rows and*$O\left(\left|D_{P}\right|\right)$*columns, such that for each meta-phrase d of*$D_{P}$*,*G*stores the positions in the* BWT *of* $P_{P}$ *where d appears.*

We now describe how to compute the characters of the BWT of *T* using these data structures. First, we compute the ranges of the BWT of *T* for the easy suffixes using only $T_{T}$ in the same manner as they were computed using traditional PFP.

Next, we show how to compute the BWT of *T* for hard-easy and hard-hard suffixes with the addition of $T_{P}$ and G. We let α be a proper phrase suffix in $S_{T}$ whose occurrences in *T* are preceded by more than one character, making it a hard suffix. We let $D_{\alpha }$ be the set of phrases of $D_{T}$ that end with α, and we consider an occurrence, say $\alpha_{i}$, as a proper phrase suffix $\alpha \in S_{T}$. We know there exists a proper meta-phrase suffix in $S_{P}$, say β, that is preceded in $P_{P}$ by only one element of $D_{\alpha }$ since α a hard-easy suffix. If we iterate over each proper meta-phrase suffix in $T_{P}$ in lexicographic order, then we know that $\alpha_{i}$ comes before all the following occurrences of α because β is lexicographically smaller than any other proper meta-phrase suffix that is preceded by an element of $D_{\alpha }$. It follows then that we know that the BWT characters preceding $\alpha_{i}$ occur before the characters preceding the next occurrences of α.

Next, we assume that while iterating over the proper meta-phrase suffixes in $S_{P}$ that are preceded by the elements of $D_{\alpha }$, there exists a proper meta-phrase suffix β in $S_{P}$ that is preceded by more than one element of $D_{\alpha }$. We consider the grid G to obtain the relative order of those occurrences of β. In particular, the ordering of the occurrences of the meta-phrases containing β in the BWT of $P_{P}$ corresponds to the ordering of the characters preceding α in the BWT of *T*.

Tables S1 and S2 give an illustration of the two tables $T_{T}$ and $T_{P}$ we build for the genomes in Figure S1. Figure S2 illustrates the grid G for the same example. We illustrate the method of constructing the BWT by computing the BWT of the genomes in Figure S1. First, we iterate over the proper phrase suffixes of $D_{T}$ stored in table $T_{T}$ in Table S1. The first suffix is preceded in the input only by the character C. Therefore, it follows that the first suffix is an easy suffix and the associated range of the BWT (i.e., [0..0]) will consist of an occurrence of the character C. The same argument holds for the second proper phrase suffix which defines the BWT range [1..3].

We now want to compute the range corresponding to the third proper phrase suffix of $D_{T}$ (i.e., $AC), namely the characters in blue and red in Figure S1. From Table S1, we know that $AC is preceded by $’s and C’s. For all phrases of $D_{T}$ in the co-lexicographic range [1..2] (i.e., the two phrases $$AC and AC$AC, respectively encoded in $P_{T}$ by the meta-characters 1 and 4), we identify all proper meta-phrase suffixes in $T_{P}$ preceded by these meta-characters. In $T_{T}$, illustrated in Figure S2, we find that the lexicographically smallest proper meta-phrase suffix, 6 12 5 20 16 23 2 $ $, is preceded in $P_{T}$ by only the meta-character 4 making this occurrence of $AC a hard-easy suffix. We can extract the corresponding character preceding $AC from the phrase corresponding to the meta-character 4 (i.e., C) by looking at $D_{T}$.

We continue iterating over the proper meta-phrase suffixes in $S_{T}$ that are preceded by either 1 or 4 and the next proper meta-phrase suffix to consider is 7 17, preceded by both the meta-characters 1 and 4, making this occurrence of $AC a hard-hard suffix. To compute the corresponding BWT characters, we consider the occurrences in the BWT of $P_{P}$ of the meta-phrases containing 7 17 in the co-lexicographic range [4..8]. From $D_{T}$, we know that the occurrences of the phrase represented by the meta-character 4 will correspond to a C in the BWT while the occurrences of 1 to a $. Following the order in which the meta-phrases appear in the BWT of $P_{P}$ stored in G and illustrated in Figure S2 in the co-lexicographic range [4..8], we can define the range [6..8] of the BWT of the input as $CC.

Oliva et al.^31^ demonstrated a method for constructing the BWT without the set of phrases using recursive PFP. However, creating the SA and LCP values was more difficult. In this work, we explain how this method can be adapted to simultaneously construct the SA and LCP alongside the BWT.

#### Computation of the SA

In order to output the SA along with the BWT, we need to be able to compute the starting position in *T* of each occurrence of a proper phrase suffix α in $S_{T}$. To accomplish this, we first revisit an observation by Kuhnle et al.^30^ that allows for the computation of the SA from PFP. Given any phrase *d* in $D_{T}$, if we let $EP_{d}$ be an array of all the ending positions of each occurrence of *d* in *T*, we can compute the SA entries. This follows from the fact that the start position of α is equal to $EP_{d}\left[j\right]-\left|\alpha \right|+1$. This strategy requires $O\left(\left|P_{T}\right|\right)$ words of memory because it requires computing and using the BWT of $P_{T}$.

##### Observation 1

*We let*$\alpha_{i}$*be any proper phrase suffix of a phrase d in*$D_{T}$*. Suppose we can define the endpoints in T of all occurrences of d in T. Then, it follows that we can define the* SA *values for all occurrences of* $\alpha_{i}$.

It follows that we can restrict interest to computing the endpoints in *T* of the occurrences of the phrases, *d*, in $D_{T}$. We will show later that we can identify these endpoints using the endpoints in *T* of the occurrences of the meta-phrases, $d^{\prime }$, in $D_{P}$.

Next, we introduce the three types of proper phrase suffixes which were previously defined by Oliva et al. in order to compute the BWT of *T*: easy, hard-easy, and hard-hard. However, in our algorithm, we consider all the proper phrase suffixes as hard-easy or hard-hard cases. This is due to the fact that, if the BWT corresponding to the occurrences of a proper phrase suffix contains a single character (i.e., easy proper phrase suffixes) then we do not need to define the order of the occurrences. However, when computing the SA values the order of the occurrences is needed to define the SA entries. Our solution relies on handling the relative order of the rotations within the range as described for the hard-hard suffixes (see Lemma 3 ^31^). Here, we show that this can be accomplished only using $D_{T}$, $D_{P}$ and $P_{P}$, eliminating the need for $P_{T}$.

##### Lemma 3

We let α be a proper phrase suffix in $S_{T}$. that is in the set of phrases $D_{\alpha }=\left\{d_{i},\ldots ,d_{n}\right\}$. We let $i_{1},..,i_{k}$ be the k occurrences of α in T then we can find the lexicographical ordering of the occurrences in $O\left(\left|D_{T}\right|+\left|D_{P}\right|+\left|P_{P}\right|\right)$-workspace.

Proof.

Given our input *T* and PFP(T). We represent the dictionary as a string $D\left[1..l\right]=d_{1}\#d_{2}\#..\#d_{m}\#$ with each dictionary phrase $d_{i}$ sorted in lexicographic order. We assume we have computed our auxiliary data structures as defined above These preliminary computations can be done in a $O\left(\left|D_{T}\right|+\left|D_{P}\right|+\left|P_{P}\right|\right)$-workspace.

Let α be any proper phrase suffix in the set $S_{T}$. We want to identify the lexicographic ordering of all substrings of *T* that start with occurrences of α. Since all these substrings start with the same |α| characters, we sort the substrings based on what follows α. First, we define the set of all phrases that contain α to be $D_{\alpha }=\left\{d_{i},\ldots ,d_{n}\right\}$, and take note of the meta-characters that represent these phrases. Then, we traverse through $T_{P}$ and identify the first proper meta-phrase suffix, say β, that is preceded by one of the meta-characters of $D_{\alpha }$. If β is preceded by a single occurrence of only one of these meta-characters, then we know that the occurrence of α contained within the meta-phrase is next in the ordering. This is a hard-easy suffix described in an earlier section. If β is instead preceded by multiple occurrences of one of these meta-characters and/or multiple of meta-characters, then we need to consider what follows β. To do this, we define $D_{\beta }=\left\{d_{i},\ldots ,d_{n}\right\}$ as the set of meta-phrases that contain β. We can then look at the grid G to identify the lexicographic ordering of these meta-phrases. As mentioned earlier, the ordering of the phrases in G is based on the lexicographic ordering of what follows them in *T*. Therefore, this is the same ordering as that of the occurrences of α.

We note that this constructive proof follows the construction of BWT from recursive PFP, which was given in Section 2.3. The main difference is that all phrases are treated as hard-easy or hard-hard suffixes. We now illustrate the proof using our previous example but extend the earlier example of the proper phrase suffix $AC, which has a total of 5 occurrences. We saw from Table S1 this proper phrase suffix can be found in the phrases whose meta-characters correspond to 1 and 4. As we traverse through Table S2, the first proper meta-phrase suffix is preceded by one of the meta-characters is 6 12 5 20 16 23 2 $ $. In this case, this proper meta-phrase suffix is preceded by a single occurrence of only 4 so there is no ambiguity and this is the first occurrence of $AC in their relative ordering. We continue to traverse Table S2 and the next proper meta-phrase suffix preceded by one of the meta-characters is 7 17. This proper meta-phrase suffix is preceded by one occurrence of 1 and three occurrences of 4. In this case, the suffix determines the next four occurrences in the ordering, but there is ambiguity to resolve. Each of these occurrences corresponds to a unique $D_{P}$ phrase which can be ordered relative to each other by using the grid G in Figure S2. We see that the meta-phrase containing 1 is first, so that meta-phrase contains the next occurrence of $AC in the ordering. Continuing in this fashion we can find the ordering of all the occurrences of $AC. Hence, by using only $D_{T}$, $D_{P}$, and $P_{P}$ we have found the lexicographical ordering of all occurrences of a proper phrase suffix in $O\left(\left|D_{T}\right|+\left|D_{P}\right|+\left|P_{P}\right|\right)$ working space.

Once we know the ordering of these occurrences, we can use the length of the phrases in $D_{T}$ and the known end positions of every occurrence of the phrases in $D_{P}$ to calculate the SA position.

##### Theorem 2

*Given a string T, the dictionary*$D_{T}$*, the parse*$P_{T}$*, the dictionary*$D_{P}$*, and the parse*$P_{P}$*obtained by running* PFP *on T and* $P_{T}$*, we can compute the* SA *using* $O\left(\left|D_{T}\right|+\left|D_{P}\right|+\left|P_{P}\right|\right)$ *working space.*

Proof.

Given our input *T* and PFP(T). We represent the dictionary as a string $D\left[1..l\right]=d_{1}\#d_{2}\#..\#d_{m}\#$ with each dictionary phrase $d_{i}$ sorted in lexicographic order. We assume that we have computed our auxiliary data structures as defined above. These preliminary computations take $O\left(\left|D_{T}\right|+\left|D_{P}\right|+\left|P_{P}\right|\right)$-time. By the properties of PFP, each suffix of *T* is prefixed by (exactly) one suffix α of a dictionary phrase $t_{j}$ with |α| is at least $w_{i}$. We call $\alpha_{i}$ the representative prefix of the suffix T[i..n]. From the uniqueness of the representative prefix, we can partition the SA into *k* ranges $\left[b_{1},e_{1}\right]$, $\left[b_{2},e_{2}\right]$,.., $\left[b_{k},e_{k}\right]$, where the $b_{i}$ and $e_{i}$ is the beginning and ending of each range. Hence, $b_{1}=1$, $b_{i}=e_{i-1}+1$ for i=2,..,k, and $e_{k}=n$, such that for i=1,..,k all suffixes in the same range (i.e., $T\left[\text{SA}\left[b_{i}\right]..n\right]$) have the same representative prefix $\alpha_{i}$. By construction, we have that $\alpha_{1}≺\alpha_{2}≺..≺\alpha_{k}$.

For each $\alpha_{i}$, we compute $\text{BWT}\left[b_{i},e_{i}\right]$ corresponding to the range $\left[b_{i},e_{i}\right]$ associated with $\alpha_{i}$ using the constructive proof for Theorem 1 It follows from Lemma 3 that we can order these occurrences of $\alpha_{i}$ within the range in $O\left(\left|P_{P}\right|+\left|D_{P}\right|+\left|D_{T}\right|\right)$ working space. Next, we find all occurrences of $\alpha_{i}$ and their ending positions in *T*. As previously mentioned, we cannot store these positions for all occurrences of phrases in $D_{T}$; instead we store the ending position of each occurrence of every meta-phrase $d^{\prime }$ in $D_{P}$ in the array $EP_{d^{\prime }}$. Next, we observe that we can calculate the length in *T* of each dictionary item in $D_{P}$. For example, suppose 1 2 3 is a dictionary item of $D_{P}$ corresponding to $d_{1}=ACCT$, $d_{2}=CTTC$, $d_{3}=TCGG$. Then the length of 1 2 3 is equal to 8.

Next, we use $T_{T}$ to find all phrases $d_{i}^{\prime }$ in $D_{P}$ with $\alpha_{i}$, and their corresponding end positions in $EP_{d_{i}^{\prime }}$. We consider the first position in $EP_{d_{i}^{\prime }}$, which we denote as *p*. We can determine the end position in *T* from *p*; by adding up the lengths of all the dictionary items before $\alpha_{i}$, we get the position where the prefix of *T* that comes before $\alpha_{i}$ ends. Let’s say the dictionary items that correspond to $d_{i}^{\prime }$ are $d_{1},d_{2},\ldots ,d_{k}$. Then, the position *p* is the sum of the lengths of all the dictionary items before $\alpha_{i}$, the lengths of the dictionary items corresponding to $d_{i}^{\prime }$, and the length of $\alpha_{i}$. This allows us to define the SA for $\alpha_{i}$. ∎

#### Computation of the LCP array

Lastly, we show that we can compute the sampled LCP array in the same workspace. Without loss of generality, hereon, we define the sampled LCP array at the first position of every run. We remind the reader that we have $EP_{d^{\prime }}$ stored for each $d^{\prime }$ in $D_{P}$; these arrays were used for the SA construction. These will be used in the proof of the following theorem.

##### Theorem 3

*The sampled* LCP *array can be constructed in* $O\left(\left|D_{T}\right|+\left|D_{P}\right|+\left|P_{P}\right|\right)$ *working space.*

Proof.

We assume that we have computed our auxiliary data structures as defined above as well as the LCP and RMQ for $P_{P}$. Additionally, we assume that we have already computed the suffix array as described above. We let *i* be the starting position of a rotation of *T*, which is preceded by a character that marks the start of a run in the BWT of *T*. We additionally let *j* be the starting position of a different rotation of *T*, such that it precedes *i* in the suffix array of *T*. By definition of PFP, there exists a prefix of the rotation of *T* starting at *i* that is a proper phrase suffix, say $\alpha_{i}$, of a phrase in $D_{T}$. It follows that there exists a prefix of the rotation of *T* starting at *j* that is also a proper phrase suffix, say $\alpha_{j}$. To compute the LCP we must compare these two rotations, which we can start to do by comparing the proper phrases suffixes they start with. If $\alpha_{i}$ and $\alpha_{j}$ are not the same proper phrase suffix, then we find the first position of mismatch and our LCP calculation is complete e.g., $\text{LCP}\left[i\right]=lcp\left(\alpha_{i},\alpha_{j}\right)$. If instead $\alpha_{i}$ and $\alpha_{j}$ are the same proper phrase suffix we must compare what follows them in the rotation.

We know that every phrase is contained in a meta-phrase and by extension so is every proper phrase suffix. As such, we can compare what follows these proper phrase suffixes by looking at the proper meta-phrase suffix that contains them. Let’s say $\alpha_{i}$ is contained by the proper meta-phrase suffix $\beta_{i}$ and $\alpha_{j}$ is contained by the proper meta-phrase suffix $\beta_{j}$. We continue the LCP computation by comparing these two proper meta-phrase suffixes. If they are different, then we find the first position of mismatch and our LCP value is the length of the proper phrase suffix $\alpha_{i}$ and the length of the proper meta-phrase suffix up until the mismatch e.g., $\text{LCP}\left[i\right]=\left|\alpha_{i}\right|+lcp\left(\beta_{i},\beta_{j}\right)$. Since the mismatch occurs at a meta-character, then we also have to compare the two phrases the meta-characters represent for correctness. We note that we have to replace each meta-character with the length of the phrase it represents to get the LCP in terms of *T*. If instead $\beta_{i}$ and $\beta_{j}$ are the same proper meta-phrase suffix, then we have to look at what follows them.

We can see what follows each meta-phrase by looking at $P_{P}$. Based on *i*, what follows the meta-phrase suffixes can include most of *T* which can be long despite being represented as the ranks of meta-phrases in $P_{P}$. To do this comparison efficiently, we use the RMQ of the LCP of $P_{P}$ to find how many of the meta-phrases that follow the meta-phrases containing $\beta_{i}$ and $\beta_{j}$ match. Once we find the meta-phrases that mismatch, we have to compare the meta-phrases to each other to further find the meta-character that mismatches within them. We then continue and compare the phrases the meta-characters represent to find the character they mismatch at. We can use the position of this mismatched character to find the LCP value.

We briefly mention that we can use a position from the SA to find the proper phrase suffix that starts at position *i* and the proper meta-phrase suffix that follows by using $EP_{d^{\prime }}$. We can use this array to find which meta-phrase contains the the position *i*. Then, we iterate through the meta-characters backwards using their expanded lengths to find which one contains *i*.∎

### Quantification and statistical analysis

We used Snakemake v9.3.0^32^ to run the experiments and used the built in benchmarking to record wall-clock time and maximum resident set size of every method on every dataset. These values were plotted directly.

## References

[1] Auton A., Brooks L.D., Durbin R.M., Garrison E.P., Kang H.M., Korbel J.O., Marchini J.L., McCarthy S., McVean G.A., Abecasis G.R., 1000 Genomes Project Consortium (2015). A global reference for human genetic variation. Nature.

[2] Turnbull C., Scott R.H., Thomas E., Jones L., Murugaesu N., Pretty F.B., Halai D., Baple E., Craig C., Hamblin A. (2018). The 100 000 Genomes Project: bringing whole genome sequencing to the NHS. Br. Med. J..

[3] Weigel D., Mott R. (2009). The 1001 Genomes Project for Arabidopsis thaliana. Genome Biol..

[4] OBrien S.J., Haussler D., Ryder O. (2014). The birds of Genome10K. GigaScience.

[5] Li H. (2013). Aligning sequence reads, clone sequences and assembly contigs with BWA-MEM. arXiv.

[6] Langmead B., Salzberg S.L. (2012). Fast gapped-read alignment with Bowtie 2. Nat. Methods.

[7] Ferragina P., Manzini G. (2005). Indexing Compressed Text. J. ACM.

[8] Burrows M., Wheeler D. (1994). Digital SRC Research Report.

[9] Sirén J., Monlong J., Chang X., Novak A.M., Eizenga J.M., Markello C., Sibbesen J.A., Hickey G., Chang P.-C., Carroll A. (2021). Pangenomics enables genotyping of known structural variants in 5202 diverse genomes. Science.

[10] Garrison E., Sirén J., Novak A.M., Hickey G., Eizenga J.M., Dawson E.T., Jones W., Garg S., Markello C., Lin M.F. (2018). Variation graph toolkit improves read mapping by representing genetic variation in the reference. Nat. Biotechnol..

[11] Rossi M., Oliva M., Langmead B., Gagie T., Boucher C. (2022). Moni: A pangenomic index for finding maximal exact matches. J. Comput. Biol..

[12] Oliva M., Rossi M., Sirén J., Manzini G., Kahveci T., Gagie T., Boucher C. (2021). 2021 Data Compression Conference (DCC).

[13] Díaz-Domínguez D., Navarro G. (2023). Efficient construction of the BWT for repetitive text using string compression. Inf. Comput..

[14] Kempa D., Kociumaka T. (2019). Proceedings of the 51st Annual ACM SIGACT Symposium on Theory of Computing.

[15] Bauer M.J., Cox A.J., Rosone G. (2013). Lightweight algorithms for constructing and inverting the BWT of string collections. Theor. Comput. Sci..

[16] Bauer M.J., Cox A.J., Rosone G. (2011). Proceedings of the 22nd Annual Symposium Combinatorial Pattern Matching.

[17] Bingmann T., Dinklage P., Fischer J., Kurpicz F., Ohlebusch E., Sanders P. (2023). Algorithms for Big Data: DFG Priority Program.

[18] Louza F.A., Telles G.P., Gog S., Prezza N., Rosone G. (2020). gsufsort: constructing suffix arrays, LCP arrays and BWTs for string collections. Algorithm Mol. Biol..

[19] Louza F.A., Gog S., Telles G.P. (2016). Proceedings of the Data Compression Conference (DCC).

[20] Liao W.-W., Asri M., Ebler J., Doerr D., Haukness M., Hickey G., Lu S., Lucas J.K., Monlong J., Abel H.J. (2023). A draft human pangenome reference. Nature.

[21] Gog S., Beller T., Moffat A., Petri M. (2014). Proc. of International Symposium on Experimental Algorithms (SEA).

[22] Louza F.A., Gog S., Telles G.P. (2017). Inducing enhanced suffix arrays for string collections. Theor. Comput. Sci..

[23] Oliva M., Cenzato D., Rossi M., Lipták Z., Gagie T., Boucher C. (2022). Proc. of IEEE Data Compression Conference (DCC).

[24] Harrison P.W., Lopez R., Rahman N., Allen S.G., Aslam R., Buso N., Cummins C., Fathy Y., Felix E., Glont M. (2021). The covid-19 data portal: accelerating sars-cov-2 and covid-19 research through rapid open access data sharing. Nucleic Acids Res..

[25] Boucher C., Cenzato D., Lipták Z., Rossi M., Sciortino M. (2021). Computing the original ebwt faster, simpler, and with less memory. arXiv.

[26] Church D.M., Schneider V.A., Steinberg K.M., Schatz M.C., Quinlan A.R., Chin C.-S., Kitts P.A., Aken B., Marth G.T., Hoffman M.M. (2015). Extending reference assembly models. Genome Biol..

[27] Gog S., Beller T., Moffat A., Petri M. (2014). *Proc. of SEA*, volume 8504 of *LNCS*.

[28] Boucher C., Gagie T., Kuhnle A., Langmead B., Manzini G., Mun T. (2019). Prefix-free parsing for building big BWTs. Algorithm Mol. Biol..

[29] Manber U., Myers G. (1993). Suffix arrays: a new method for on-line string searches. SIAM J. Comput..

[30] Kuhnle A., Mun T., Boucher C., Gagie T., Langmead B., Manzini G. (2020). Efficient construction of a complete index for pan-genomics read alignment. J. Comput. Biol..

[31] Oliva M., Gagie T., Boucher C. (2023). IEEE Data Compression Conference (DCC).

[32] Mölder F., Jablonski K.P., Letcher B., Hall M.B., Tomkins-Tinch C.H., Sochat V., Forster J., Lee S., Twardziok S.O., Kanitz A. (2021). Sustainable data analysis with Snakemake. F1000Research.

