# Supplementary material for: Building a pangenome alignment index via recursive prefix-free parsing
Source: iScience. 2024 Sep 12;27(10):110933. doi: 10.1016/j.isci.2024.110933 (PMC11465122; doi:10.1016/j.isci.2024.110933)
Supplement: Document S1. Figures S1 and S2 and Tables S1 and S2 [file mmc1.pdf]

iScience, Volume *27*

Supplemental information

Building a pangenome alignment index via recursive prefix-free parsing

Eddie Ferro, Marco Oliva, Travis Gagie, and Christina Boucher

```
T=ACGTTCGCAACTAGTCCGGGAGTTAC$ACGTTCGGAATAGTTCCGGGAGGTTAAC$
  AACTTCGTCACCTAGTCCGGGAGGTAAC$AACTTCGCACCTAGTCCGGGACGTTTAC$
  ACGTTCGTCACTAGTTCCGGGAGTTAC$AACTTCGGAATAGTCCGGGACTAAC$
  ACGTTCGTGACTAGTTCCGGGACTAAC$ACCTTCGCACCTAGTCCGGGAGTTAC$$
```

F	M	BWT
$	...	C
$	...	C
$	...	C
$	...	C
$ACC	...	ACTAAC
$ACGTTCGC	...	AC$$$
$ACGTTCGG	...	GTTAC
$ACGTTCGTC	...	TTTAC
$ACGTTCGTG	...	CTAAC
⋮	⋮	⋮
ACGTTCGCAACTAGT	...	CCGGGAGTTAC$$
⋮	⋮	⋮
T	...	G
T	...	G
T	...	G

Figure S1: An illustration of a set of genomic sequences and their . At the top is a set of genomic sequences that are concatenated together and delimited by $'s, with the final haplotyped delimited by $$. On the bottom is the BWT matrix (M) of of the concatenated sequence. The last column of the matrix is the BWT. For ease of the illustration, we do not show the entire matrix or BWT. We use the range $[4..8]$ as our ongoing example in this paper. The characters in the range $[0..3]$ correspond to easy suffixes, the character in cyan to a hard-easy suffix, and the characters in red to hard-hard suffixes, related to STAR Methods.

0	7 17 25 17 21 9 24 14 8 4 6 12 5 20 16 23 2 $ $
1	$ $ 1
2	3 10 17 19 5 20 15 18 3 10
3	7 17 25 13 24 15 22 3 10
4	7 17 25 17 19 9 24 16 23 3 10
5	$ 1 7 17
6	7 17 25 11 9 20 16 23 4 7 17
7	7 17 26 4 7 17
8	3 10 13 20 14 8 4 7 17
9	3 10 12 5 20 14 7 17

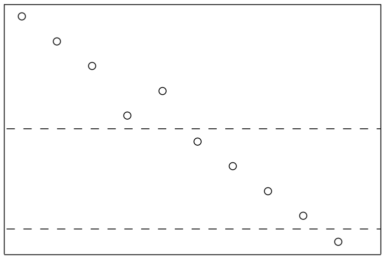

Figure S2: Our grid $\mathcal{G}_2$ showing how the phrases (in co-lexicographic order of the expanded phrases) occur in the BWT of P_2, related to STAR Methods.

phrase suffix	BWT range	co-lex range	co-lex sub-ranges `$`	`A`	`C`	`G`	`T`
`$$`	0–0	0–0			0–0		
`$AAC`	1–3	3–3			3–3		
`$AC`	4–8	1–2	1–1		2–2		
`AAC`	9–16	3–7	3–3		4–4		5–7
`AATAGT`	17–18	21–21				21–21	
`AC`	19–43	1–13	1–2	3–7	8–9	10–11	12–13
`AGGT`	44–45	25–25				25–25	
`AGT`	46–56	20–23				20–20	21–23
`ATAGT`	57–58	21–21		21–21			
`C$$`	59–59	0–0		0–0			
`C$AAC`	60–62	3–3		3–3			
`C$AC`	63–66	2–2		2–2			
`CAAC`	67–67	4–4				4–4	
`CAC`	68–71	8–9				8–8	9–9
`CCG`	72–79	15–16					15–16
`CCTAGT`	80–82	23–23		23–23			
`CCTTCG`	83–83	18–18		18–18			
`CG`	84–104	14–19		14–14	15–16		17–19
`CTAAC`	105–106	5–5		5–5			
`CTAGT`	107–112	22–23		22–22	23–23		
`CTTCG`	113–116	17–18		17–17	18–18		
`GAATAGT`	117–118	21–21				21–21	
`GAC`	119–122	10–11				10–10	11–11
`GAGGT`	123–124	25–25				25–25	
`GAGT`	125–127	20–20				20–20	
`GCAAC`	128–128	4–4			4–4		
`GCAC`	129–130	8–8			8–8		
`GGAATAGT`	131–132	21–21			21–21		
`GGAC`	133–135	10–10				10–10	
`GGAGGT`	136–137	25–25				25–25	
`GGAGT`	138–140	20–20				20–20	
`GGGAC`	141–143	10–10			10–10		
`GGGAGGT`	144–145	25–25			25–25		
`GGGAGT`	146–148	20–20			20–20		
`GGT`	149–150	25–25		25–25			
`GT`	151–171	20–25		20–23	24–24	25–25	
`TAAC`	172–175	5–7			5–5	6–6	7–7
`TAC`	176–179	12–13					12–13
`TAGT`	180–187	21–23		21–21	22–23		
`TCAC`	188–189	9–9				9–9	
`TCCG`	190–197	15–16				15–15	16–16
`TCG`	198–205	17–19					17–19
`TGAC`	206–206	11–11				11–11	
`TTAAC`	207–207	7–7				7–7	
`TTAC`	208–211	12–13				12–12	13–13
`TTCCG`	212–214	16–16				16–16	
`TTCG`	215–222	17–19			17–18	19–19	
`TTTAC`	223–223	13–13				13–13	

Table S1: Our table $\mathcal{T}_T$ storing all proper phrase suffixes of D_T of length at least $w_1 = 2$, related to STAR Methods.

phrase suffix	co-lex range	preceding meta-characters
$ $	0–0	2
$ 1	4–4	$
1 7 17	5–5	$
2 $ $	0–0	23
3 10	1–3	18,22,23
4 6 12 5 20 16 23 2 $ $	0–0	8
4 7 17	6–8	8,23,26
5 20 14 7 17	9–9	12
5 20 15 18 3 10	3–3	19
5 20 16 23 2 $ $	0–0	12
6 12 5 20 16 23 2 $ $	0–0	4
7 17	5–9	1,14,4,4,4
8 4 6 12 5 20 16 23 2 $ $	0–0	14
8 4 7 17	8–8	14
9 20 16 23 4 7 17	6–6	11
9 24 14 8 4 6 12 5 20 16 23 2 $ $	0–0	21
9 24 16 23 3 10	2–2	19
10 12 5 20 14 7 17	9–9	3
10 13 20 14 8 4 7 17	8–8	3
10 17 19 5 20 15 18 3 10	3–3	3
11 9 20 16 23 4 7 17	6–6	25
12 5 20 14 7 17	9–9	10
12 5 20 16 23 2 $ $	0–0	6
13 20 14 8 4 7 17	8–8	10
13 24 15 22 3 10	1–1	25
14 7 17	9–9	20
14 8 4 6 12 5 20 16 23 2 $ $	0–0	24
14 8 4 7 17	8–8	20
15 18 3 10	3–3	20
15 22 3 10	1–1	24
16 23 2 $ $	0–0	20
16 23 3 10	2–2	24
16 23 4 7 17	6–6	20
17 19 5 20 15 18 3 10	3–3	10
17 19 9 24 16 23 3 10	2–2	25
17 21 9 24 14 8 4 6 12 5 20 16 23 2 $ $	0–0	25
17 25 11 9 20 16 23 4 7 17	6–6	7
17 25 13 24 15 22 3 10	1–1	7
17 25 17 19 9 24 16 23 3 10	2–2	7
17 25 17 21 9 24 14 8 4 6 12 5 20 16 23 2 $ $	0–0	7
17 26 4 7 17	7–7	7
18 3 10	3–3	15
19 5 20 15 18 3 10	3–3	17
19 9 24 16 23 3 10	2–2	17
20 14 7 17	9–9	5
20 14 8 4 7 17	8–8	13
20 15 18 3 10	3–3	5
20 16 23 2 $ $	0–0	5
20 16 23 4 7 17	6–6	9
21 9 24 14 8 4 6 12 5 20 16 23 2 $ $	0–0	17
22 3 10	1–1	15
23 2 $ $	0–0	16
23 3 10	2–2	16
23 4 7 17	6–6	16
24 14 8 4 6 12 5 20 16 23 2 $ $	0–0	9
24 15 22 3 10	1–1	13
24 16 23 3 10	2–2	9
25 11 9 20 16 23 4 7 17	6–6	17
25 13 24 15 22 3 10	1–1	17
25 17 19 9 24 16 23 3 10	2–2	17
25 17 21 9 24 14 8 4 6 12 5 20 16 23 2 $ $	0–0	17

Table S2: Our table $\mathcal{T}_P$ storing all proper phrase suffixes of D_P of length at least $w_2 = 2$, related to STAR Methods.
